# Surgical Uses of Linen Thread

**Published:** 1902-05-31

**Authors:** 


### SURGICAL USES OF LINEN THREAD.

Substances used in the arts may often be usefully applied to surgical purposes, and in many cases they possess the great advantages of being far cheaper and much more easily procurable than analogous materials specially prepared for use in surgery. In this connection we would refer to Mr. Arthur Barker's[1] recommendation of the use of linen sewing-machine thread for ligatures and sutures. This material is quite different from the hempen thread which in old days was commonly employed for ligatures, and of course it must not be confounded with sewing-machine cotton, which is quite another thing. It possesses many good points. It can be procured everywhere all over the world wherever there is a depôt for Singer's sewing machines; it is relatively very cheap; it can easily be sterilised by boiling in plain water and then stored in methylated spirit; it is enormously strong, and ties a most uncompromising knot; it is easy to work with, and runs through the eye of any suitable needle easily, having been spun with special care in order to travel evenly through the sewing-machine needle. The following three sizes will be found the most useful:—No. 40, which is as thick as need be desired for the abdominal wall or the ligature of the larger arteries; No. 60, which is thinner, but still very strong; and No. 90, which is as fine as can be desired, say, for a suture of the intestine. Mr. Barker finds it convenient to procure No. 40 as a white thread, No. 60 in red, and No. 90 in black. The thread can be boiled over and over again without rotting it.

[1] Lancet, May 24.

